# Cardiac Imaging in the Diagnosis and Management of Heart Failure

**DOI:** 10.3390/jcm14145002

**Published:** 2025-07-15

**Authors:** Mayuresh Chaudhari, Mahi Lakshmi Ashwath

**Affiliations:** 1 UT Health Sciences Centre, Glen Biggs Institute, San Antonio, TX 78240, USA; 2 UHS/UT Heart and Vascular Institute, San Antonio, TX 78240, USA

**Keywords:** echocardiography, cardiac magnetic resonance imaging (MRI), myocardial viability, tissue characterization, ischemic and non-ischemic cardiomyopathy, heart failure with preserved ejection fraction, right heart failure, left ventricular failure, myocardial strain

## Abstract

Heart failure (HF) is a complex clinical syndrome that results from any structural or functional impairment of ventricular filling or ejection of blood. The etiology of heart failure is multifactorial, encompassing ischemic heart disease, hypertension, valvular disorders, cardiomyopathies, and metabolic and infiltrative diseases. Despite advances in pharmacologic and device-based therapies, heart failure continues to carry a substantial burden of morbidity, mortality, and healthcare utilization. With the advancement and increased accessibility of cardiac imaging modalities, the diagnostic accuracy for identifying the underlying etiologies of nonischemic cardiomyopathy has significantly improved, allowing for more precise classification and tailored management strategies. This review aims to provide a comprehensive analysis of the current understanding of heart failure, encompassing epidemiology, etiological factors, with a specific focus on diagnostic imaging modalities including the role of echocardiography and strain imaging, cardiac magnetic resonance imaging (CMR), cardiac computed tomography (CT), and nuclear positron emission tomography (PET) imaging and recent advances in the diagnosis and management of heart failure.

## 1. Introduction

Heart failure (HF) is a complex clinical condition that results from any structural or functional impairment of ventricular filling or ejection of blood. Most common presentations include dyspnea, fatigue, and fluid retention, which can significantly impair quality of life and lead to frequent hospitalizations. Most cardiovascular diseases culminate into heart failure as a terminal event. It is a rising global health problem, with increasing prevalence due to aging populations, improved survival due to prompt medical, interventional and device therapies, and optimal management of chronic cardiovascular conditions [1,2].

Based on the left ventricular ejection fraction (LVEF), heart failure is classified into two main phenotypes:

Heart failure with reduced ejection fraction (HFrEF) typically defined as LVEF < 40%, where systolic dysfunction predominates.

Heart failure with preserved ejection fraction (HFpEF)—with LVEF ≥ 50%, primarily involving diastolic dysfunction, impaired ventricular relaxation, and increased stiffness.

A third intermediate category, heart failure with mildly reduced ejection fraction (HFmrEF), usually defined as LVEF 41–49%, has been recognized more recently, reflecting the clinical and therapeutic overlap between HFrEF and HFpEF [1].

The etiology of HF includes several common conditions like ischemic heart disease, hypertension, valvular disorders, cardiomyopathies, and metabolic and infiltrative diseases. Non-cardiac comorbidities—such as diabetes mellitus, chronic kidney disease, and obesity—also play a pivotal role in the progression and prognosis of HF. Reversible causes like anemia, hyperthyroidism, and excessive alcohol consumption also contribute to heart failure.

Despite advances in pharmacologic and device-based therapies, HF still carries a substantial impact on morbidity, mortality, and healthcare utilization. Recent advances and emerging therapeutic strategies for HFrEF include the use of angiotensin receptor-neprilysin inhibitors (ARNIs) and sodium-glucose cotransporter-2 inhibitors (SGLT2is) in addition to evidence-based medications such as beta-blockers and mineralocorticoid receptor antagonists (MRAs), considered the ‘four pillars’ of heart failure treatment. Therapeutic choices are more limited for HFpEF, but recent studies have shown promise for some pharmacological agents in improving outcomes.

Myocardial viability and the concept of hibernating myocardium remain important and debated topics in heart failure, particularly in patients with ischemic cardiomyopathy. Viable yet chronically under-perfused myocardium (hibernating myocardium) may retain the potential for functional recovery if revascularized. Imaging modalities like dobutamine stress echocardiography, cardiac MRI with late gadolinium enhancement, and PET are used to assess viability. However, recent trials (e.g., STICH) have challenged the routine use of viability testing to guide revascularization, suggesting that its impact on long-term outcomes may be limited. Despite this, viability assessment can still be valuable in select patients, particularly when decisions about advanced therapies or revascularization are uncertain.

Diagnostic limitations in heart failure often stem from the inability to accurately assess cardiac structure, function, and hemodynamics using clinical evaluation alone. Imaging technologies, such as echocardiography, cardiac MRI, and CT, are intended to overcome these limitations by providing detailed visualization of ventricular function, chamber size, wall motion abnormalities, valvular pathology, and myocardial tissue characterization—facilitating earlier diagnosis, more precise classification (e.g., HFpEF vs. HFrEF), and tailored treatment strategies.

Given the heterogeneity of HF and its dynamic clinical course, ongoing research continues to refine diagnostic tools, biomarkers, risk stratification models, and personalized therapeutic approaches. This review aims to provide a comprehensive analysis of the current understanding of heart failure, encompassing epidemiology, etiological factors, diagnostic imaging modalities including role of echocardiography and strain imaging, cardiac magnetic resonance imaging (CMR), cardiac computed tomography and nuclear positron emission tomography (PET) imaging, and emerging research directions in the diagnosis and management of HF.

## 2. Epidemiology of Heart Failure

Heart failure (HF) is a significant health issue in the US with rising incidence and prevalence across both developed and developing nations. It affects over 64 million people worldwide, with estimates continuing to increase due to aging populations, prolonged survival following acute coronary events, and improved management of cardiovascular diseases. In high-income countries, the lifetime risk of developing HF is estimated at 20–33% after the age of 40, with men and women affected almost equally, although clinical characteristics and outcomes differ by sex [2].

About 6.7 million adults are suffering from HF in the US, a figure forecasted to surpass 8 million by 2030. Prevalence varies from 1% to 2% of the adult population in Europe to exceeding 10% in people over 70 years of age [2,3].

Though the epidemiological profile can vary because of distinctive causes like rheumatic heart disease, untreated hypertension, and infectious cardiomyopathies, HF is also becoming a major source of mortality and disability in low- and middle-income countries.

In some developed countries, the rate of HF has stayed fairly constant or somewhat decreased due in part to improved control of risk factors like hypertension and coronary artery disease (CAD). Nevertheless, patients with HF continue to be significant drivers of healthcare costs, particularly among older adults. In older U.S. patients 65 years and above and in many European nations, HF is the leading cause of hospitalization [2,3].

Epidemiologically, heart failure can be classified into its subtypes:

HFrEF is more commonly associated with male sex, prior history of myocardial infarction, and reduced left ventricular function.

HFpEF is more prevalent among elderly women, with comorbidities such as obesity, diabetes, atrial fibrillation, and hypertension.

**Figure 1:**
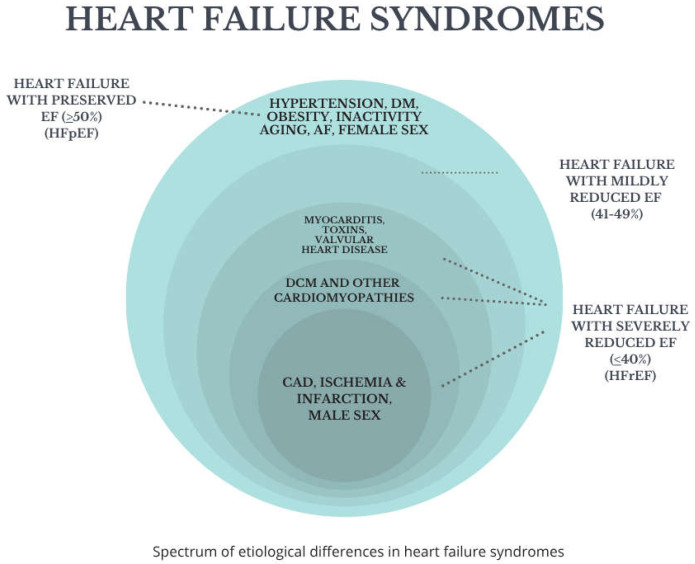
Spectrum of heart failure syndromes.

HFmrEF occupies a middle ground, sharing features of both HFrEF and HFpEF, though it is less well characterized in population studies (Figure 1).

Mortality remains high despite therapeutic advancements. Five-year mortality rates following diagnosis of HF range from 40% to 60%, comparable to many malignancies. Moreover, recurrent hospitalizations are associated with progressively worse outcomes, refractory to therapy and ultimately increased risk of death [2,3].

Disparities in HF prevalence and outcomes are also evident based on socioeconomic status, race, and geographic location. For example, Black individuals in the U.S. have a higher incidence of HF at younger ages, often with more severe disease and worse outcomes, influenced by a complex interplay of genetics, comorbidities, access to care, and social determinants of health [3,4].

As HF continues to impose a growing clinical and economic burden globally, a thorough knowledge of its epidemiology is crucial for health systems planning, resource distribution, and implementing preventive strategies (Table 1).

## 3. Etiology of Heart Failure

**Figure 2:**
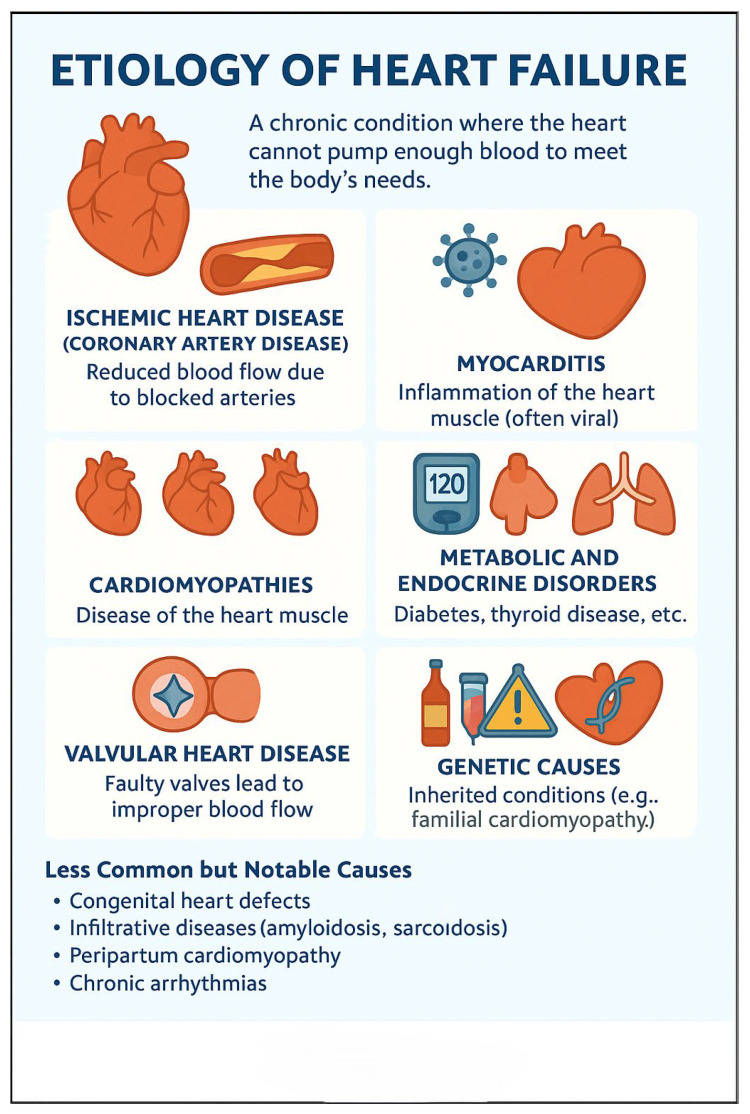
Etiology of heart failure.

The etiology of HF is multifactorial, including ischemic heart disease, hypertension, valvular disorders, cardiomyopathies, peri-partum, and metabolic and infiltrative diseases (Figure 2). Non-cardiac comorbidities such as diabetes mellitus, chronic kidney disease, obesity, and anemia also play a pivotal role in the progression and prognosis of HF (Table 2).

## 4. Diagnosis of Heart Failure

The diagnosis of HF involves a multifaceted approach that integrates clinical evaluation, laboratory testing, and imaging to confirm the presence of cardiac dysfunction, evaluate severity, and identify underlying etiology.

Clinical assessment remains the cornerstone of diagnosis. Patients typically present with nonspecific symptoms such as dyspnea, fatigue, and exercise intolerance. Dyspnea may occur on exertion, while more advanced cases report orthopnea and paroxysmal nocturnal dyspnea. Peripheral edema, weight gain, and abdominal distension suggest fluid retention [21]. On physical examination, findings such as elevated jugular venous pressure (JVP), pulmonary rales, third heart sound (S3), hepatomegaly, and peripheral edema support the diagnosis [22].

Laboratory investigations are essential in the diagnostic workup. Measurement of natriuretic peptides, specifically B-type natriuretic peptide (BNP) and N-terminal pro-BNP (NT-pro BNP), is highly recommended. Elevated levels of these peptides are strongly suggestive of heart failure and are especially helpful in differentiating cardiac from non-cardiac causes of dyspnea [23]. Routine tests include complete blood count, renal and liver function tests, thyroid function (TSH), serum electrolytes, and blood glucose to detect concomitant diseases or possible contributory factors [24,25].

Imaging, particularly transthoracic echocardiography, plays a pivotal role. It provides direct evaluation of left ventricular systolic and diastolic function, identifies wall motion abnormalities, and detects valvular heart disease. It also enables classification into HFrEF, HFpEF, or HFmrEF [26,27]. A chest radiograph may reveal cardiomegaly, pulmonary venous congestion, interstitial edema, or pleural effusions, though these findings are not specific [28].

An electrocardiogram (ECG) is routinely performed and may reveal evidence of prior myocardial infarction, left ventricular hypertrophy, arrhythmias (e.g., atrial fibrillation), or conduction abnormalities such as bundle branch blocks, all of which support a cardiac etiology [29].

**Figure 3:**
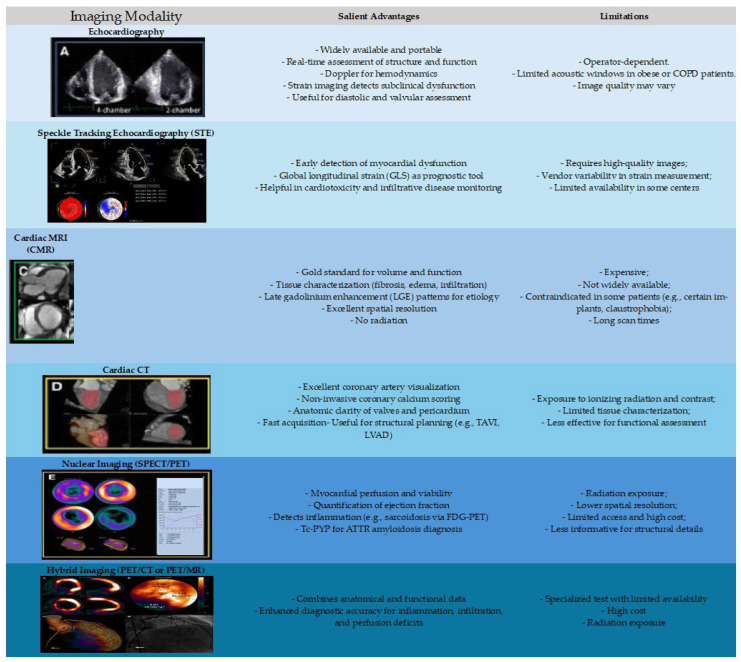
Heart failure assessment with available imaging modalities.

Advanced diagnostic tools could be needed in certain cases. A summary of key imaging modalities has been depicted in Table 3 and Figure 3. Cardiac magnetic resonance imaging (MRI) provides high-resolution assessment of myocardial structure, function, and tissue characterization, which is particularly valuable in cases of suspected myocarditis, infiltrative cardiomyopathies, or iron overload [30]. Catheter coronary angiography or non-invasive ischemia testing using coronary CTA is considered when ischemic heart disease is suspected. Right heart catheterization is useful to assess hemodynamic status in cases with diagnostic uncertainty or in patients with advanced or refractory symptoms [31].

Heart failure with preserved ejection fraction (HFpEF) presents unique imaging-related challenges, as traditional measures like left ventricular ejection fraction (LVEF) remain normal, making diagnosis less straightforward. Variability in diagnostic criteria, operator dependence, and limited accessibility to advanced modalities continue to complicate consistent evaluation and management. In HFpEF, abnormalities are often in diastolic function and myocardial mechanics, which are not detectable through conventional echocardiography or LVEF assessment alone. Diastolic dysfunction, a hallmark of HFpEF, is complex to evaluate. Doppler echocardiography indices (e.g., E/A ratio, E/e’ ratio, left atrial volume) can be load-dependent and variable, reducing diagnostic accuracy. HFpEF encompasses multiple phenotypes (e.g., hypertensive heart disease, obesity-related HFpEF, infiltrative cardiomyopathies), each with distinct imaging patterns, making a one-size-fits-all imaging strategy ineffective. Advanced imaging techniques such as strain imaging and cardiac MRI require specialized expertise and equipment, which may not be widely available in all clinical settings. Speckle-Tracking Echocardiography (STE) assesses global longitudinal strain (GLS), which can detect subclinical systolic dysfunction in patients with preserved EF. It is more sensitive than LVEF and is increasingly used to identify early myocardial impairment. Evaluating cardiac function during exercise or pharmacologic stress during diastolic stress echocardiography can unmask abnormal filling pressures that are not evident at rest. This is particularly useful in patients with exertional symptoms but inconclusive resting studies. CMR offers high-resolution assessment of myocardial tissue characteristics. Late gadolinium enhancement (LGE) and T1 mapping can detect fibrosis, infiltration (e.g., amyloidosis), or inflammation—features often present in HFpEF phenotypes. As the left atrium reflects chronic diastolic burden, strain imaging of the LA can provide prognostic information and improve diagnostic specificity for HFpEF. Tailored multimodality imaging approaches incorporating routine echocardiography with CMR or cardiac CT, particularly in challenging cases, allow better characterization of comorbidities such as coronary artery disease, pericardial disease, or pulmonary hypertension, which often coexist with HFpEF, thereby supporting accurate diagnosis and guiding management in this complex yet rampant patient population [22].

**Figure 4:**
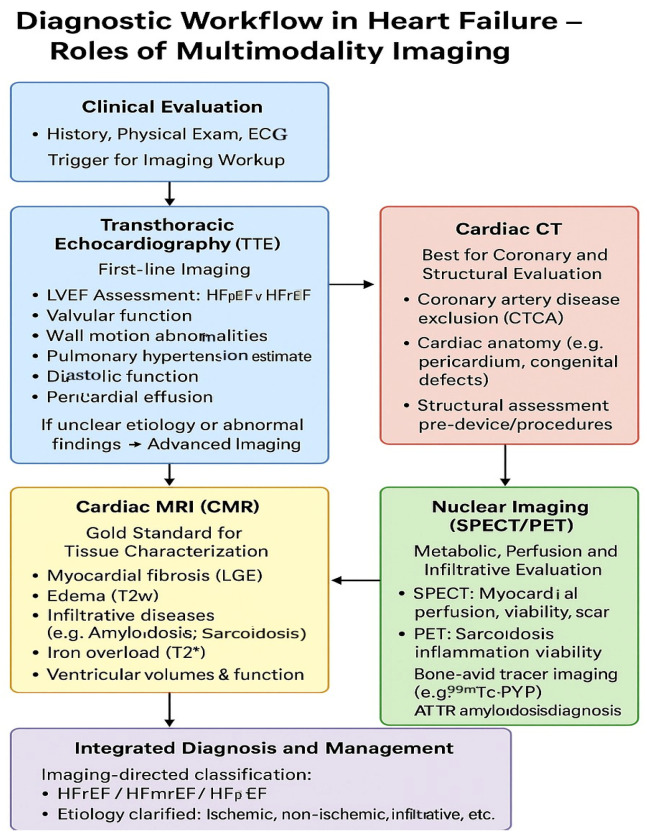
Diagnostic workflow in heart failure—role of multimodality imaging.

Early and accurate diagnosis of heart failure is extremely vital for prompt initiation of evidence-based therapies and improving clinical outcomes (Figure 4).

## 5. Role of Echocardiography and Strain Imaging in Heart Failure

Echocardiography is the primary imaging modality for the evaluation and management of patients with HF. It is a widely available, non-invasive, and cost-effective bedside test providing essential information about cardiac structure, function, and hemodynamics. Transthoracic echocardiography (TTE) is recommended as the first-line imaging study in all patients with suspected HF and for serial follow-up in confirmed HF patients to assess response to therapy [32].

Echocardiography allows for the assessment of left ventricular ejection fraction (LVEF), which is critical for classifying HF into the three categories [33]. LVEF also guides therapeutic decisions, including the use of beta-blockers, ACE inhibitors/ARNI, MRAs, and device therapy [34]. In addition, echocardiography evaluates chamber sizes, wall thickness, valvular function, diastolic function, and pericardial disease, providing a comprehensive view of cardiac anatomy and physiology.

In recent years, strain imaging, particularly global longitudinal strain (GLS) using speckle-tracking echocardiography (STE), has emerged as a powerful tool for detecting subclinical myocardial dysfunction. GLS quantifies myocardial deformation during systole and is more sensitive than LVEF in identifying early myocardial impairment [35]. It is especially useful in patients with HFpEF, where LVEF remains normal despite significant functional limitation [36], and in situations when CMR is not feasible. Patients with symptoms of HF and preserved LV systolic function on echocardiography should undergo detailed assessment of diastolic function using Doppler echocardiography and strain imaging (Table 4 and Table 5).

Echocardiographic and strain imaging criteria for diastolic heart failure (HFpEF):

Supportive echo findings for HFpEF diagnosis include the following [39,40].

**Figure 5:**
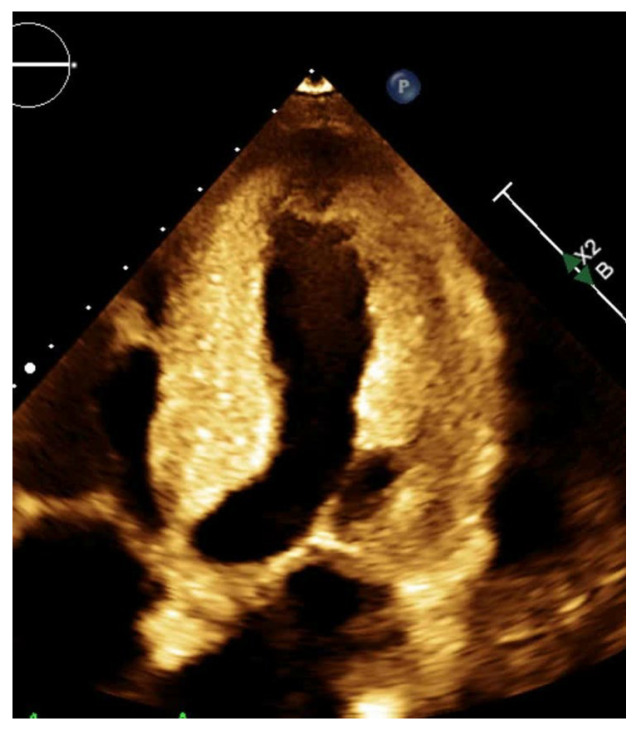
2D Echocardiography apical 4 chamber view depicting significant concentric LV hypertrophy with speckled or granular appearance in a patient with cardiac amyloidosis.

- Preserved LVEF (≥50%);
- Increase in LV wall thickness (e.g., concentric hypertrophy) (Figure 5);
- Enlarged Left atrium (due to chronic elevated pressures);
- Normal or mildly reduced GLS;
- Diastolic dysfunction grade (≥2).

It is important to look for uncommon possibilities like apical hypertrophic cardiomyopathy (apical HCM) and endomyocardial fibrosis (EMF) in the differential diagnosis of HFpEF. Apical HCM is characterized by localized thickening of the left ventricular apex, often producing a “spade-like” configuration of the LV cavity on imaging. On echocardiography, it may show isolated apical hypertrophy without LV outflow tract obstruction, and contrast echo using agents like Definity or Optison may be required to visualize the apex clearly. It is commonly associated with diastolic dysfunction and deep T-wave inversions on ECG. CMR is often used to confirm the diagnosis, as it provides superior apical visualization and can assess for late gadolinium enhancement (fibrosis) [39,40].

EMF, by contrast, is characterized by fibrosis of the endocardium and sub endocardium, especially affecting the apices of the ventricles. It often leads to cavity obliteration, restrictive physiology, and sometimes thrombus formation. Unlike infiltrative cardiomyopathies, the myocardium itself is not thickened [39,40].

Strain imaging also provides prognostic information. Studies have demonstrated that reduced GLS is independently associated with adverse outcomes in both HFrEF and HFpEF populations [41]. Furthermore, GLS is valuable in monitoring cardiotoxicity in patients undergoing chemotherapy and detecting early myocardial involvement in infiltrative diseases such as amyloidosis or sarcoidosis [42,43]. GLS abnormalities may precede LVEF decline by weeks to months, allowing early intervention (e.g., ACE inhibitors, beta-blockers) to prevent irreversible damage (Table 6).

Additionally, right ventricular (RV) strain and left atrial strain measurements offer incremental value in assessing RV function and atrial remodeling, both of which are key prognostic indicators in heart failure [47].

Strain imaging is a powerful adjunct, especially for early disease and prognostic assessment, but not a standalone diagnostic criterion for heart failure per current ESC and ACC/AHA guidelines [21,22]. Strain can complement these findings but cannot replace the clinical criteria for diagnosis of HF (symptoms, signs, natriuretic peptides, assessment of EF, structural/functional abnormalities). Salient characteristic echo and strain imaging features in different etiologies of nonischemic cardiomyopathy have been highlighted in the following table (Table 7).

Echocardiographic and Strain Imaging Criteria for Right Heart Failure and Pulmonary Arterial Hypertension (PAH):

Right heart failure is typically caused by pressure overload (e.g., from PAH), volume overload, or intrinsic RV dysfunction. It manifests as systemic congestion and reduced cardiac output (Table 8).

PAH is defined by mean pulmonary artery pressure (mPAP) > 20 mmHg, PA wedge pressure ≤ 15 mmHg, and PVR > 2 Wood units. ECHO serves as a screening and monitoring tool (Table 9).

RV strain is more sensitive than TAPSE or FAC for early detection of RV dysfunction and is prognostic in both PAH and RHF [51,52] (Table 10).

Assessment of myocardial viability is clinically relevant in patients with ischemic heart failure, particularly when considering revascularization strategies. The concept centers on identifying hibernating myocardium—chronically dysfunctional but viable tissue that may recover function after restoring blood flow. Several imaging modalities are employed to evaluate viability, each with distinct mechanisms and clinical utility [53,54].

Dobutamine stress echocardiography assesses contractile reserve by evaluating wall motion response to low-dose dobutamine. Improvement in contractility indicates viable myocardium. This method is widely available and cost-effective but is limited by image quality and operator dependence.

In summary, echocardiography remains the cornerstone for heart failure assessment, while strain imaging enhances diagnostic sensitivity, risk stratification, and early detection of myocardial dysfunction. Together, they form a comprehensive, dynamic imaging strategy for the evaluation and management of heart failure.

## 6. Role of Cardiac Magnetic Resonance Imaging in Heart Failure

Cardiac magnetic resonance imaging (CMR) is an advanced non-invasive modality that is increasing becoming a frontrunner in the evaluation and management of heart failure (HF), particularly in nonischemic cardiomyopathy, where echocardiographic findings are inconclusive, or further tissue characterization is required. CMR provides accurate, reproducible assessment of ventricular volumes, mass, and ejection fraction and is considered the gold standard for quantifying left and right ventricular function [53].

One of the most powerful features of CMR is its ability to perform tissue characterization through techniques such as late gadolinium enhancement (LGE) and parametric mapping. LGE enables detection of myocardial fibrosis and scar, offering insight into etiology. For example, subendocardial or transmural LGE patterns suggest ischemic cardiomyopathy, while mid-wall or patchy LGE is characteristic of non-ischemic etiologies such as dilated cardiomyopathy or myocarditis [54,55].

Cardiac magnetic resonance imaging (CMR) with late gadolinium enhancement (LGE) is considered the gold standard for scar quantification. It differentiates viable from non-viable myocardium based on contrast uptake: subendocardial or transmural LGE indicates fibrosis, with a transmural extent greater than 50% correlating with poor likelihood of recovery.

CMR is increasingly useful in diagnosing infiltrative cardiomyopathies. In cardiac amyloidosis, a global subendocardial or transmural LGE with difficulty nulling the myocardium is typical, while T1 mapping shows elevated native T1 and extracellular volume (ECV) [56]. In Anderson–Fabry disease, LGE is often confined to the basal inferolateral wall, and T1 mapping shows reduced native T1 values [57]. CMR also aids in evaluating other etiologies like sarcoidosis, hemochromatosis, hypertrophic cardiomyopathy, and arrhythmogenic right ventricular cardiomyopathy (ARVC), which recently have been diagnosed more frequently with the increasing usage of CMR [58] (Table 11).

In myocarditis, CMR fulfills the Lake Louise Criteria, using a combination of T2-weighted imaging, early gadolinium enhancement, and LGE to confirm inflammation and necrosis [59]. It can also detect microvascular obstruction and intramyocardial hemorrhage in the setting of acute myocardial infarction, which has prognostic significance.

CMR is particularly helpful in HF with preserved ejection fraction (HFpEF) when the etiology is uncertain, as it can uncover subtle myocardial abnormalities not apparent on echocardiography. It is also used for serial follow-up of myocardial remodeling or cardiotoxicity in patients undergoing chemotherapy [60].

Despite its advantages, CMR has limitations, including inability to image in patients with claustrophobia, contraindications in patients with certain metallic implants, cost, and limited availability in some settings. However, its high diagnostic yield and multiparametric capabilities make it an invaluable tool in the modern heart failure diagnostic arsenal.

**Table 11:** Characteristic cardiac MRI findings in heart failure etiologies (Figure 6 and Figure 7) [55,56,57,58,59].

Condition	Late Gadolinium Enhancement (LGE)	T1 Mapping/ECV	Other Key CMR Features
Ischemic Cardiomyopathy	Subendocardial or transmural in coronary artery distribution	↑ native T1 and ECV in infarcted zones	Wall thinning, scar extent correlates with prognosis
Dilated Cardiomyopathy	Mid-wall septal or diffuse patchy	Mild ↑ T1 and ECV	Chamber dilation, reduced EF, global hypokinesia
Hypertrophic Cardiomyopathy	Focal patchy LGE at RV insertion points or mid-wall	↑ native T1 and ECV (variable)	Asymmetric hypertrophy, LVOT obstruction, apical variant
Cardiac Amyloidosis	Global subendocardial or transmural; difficulty nulling myocardium	Markedly ↑ native T1 and ECV	Biatrial enlargement, thickened valves/pericardium, small LV
Anderson-Fabry Disease	Focal inferolateral wall LGE	↓ native T1	Concentric hypertrophy, no ECV increase
Myocarditis	Subepicardial or mid-wall (especially lateral wall)	↑ native T1 and T2	Edema on T2, positive Lake Louise criteria
Sarcoidosis	Patchy, multifocal LGE (basal septum ‘hook sign’ often seen	↑ native T1 and ECV	RV involvement, wall thinning, edema possible
Iron Overload (Hemochromatosis)	Usually none unless fibrosis is advanced	↓ native T1	Reduced T2* signal (<20 ms diagnostic), biventricular dilation
Arrhythmogenic RV Cardiomyopathy (ARVC)	RV free wall LGE (if visible)	Variable	RV dilation, akinesia, fatty infiltration (on black-blood imaging)

**Figure 6:**
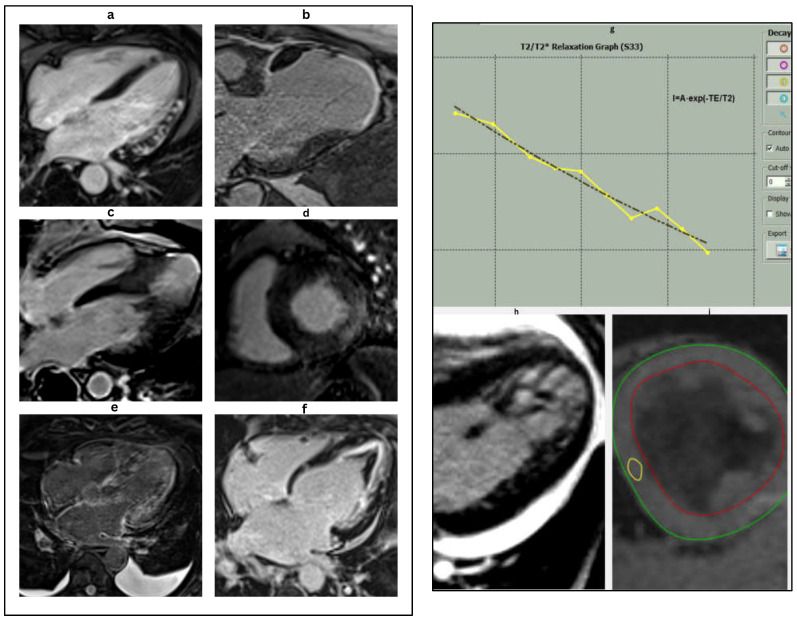
Characteristic cardiac MRI findings in different HF etiologies. (**a**): Four chamber delayed enhancement CMR in a patient with myocarditis. Late gadolinium enhancement (LGE) is seen in the mid-myocardial to subepicardial layers of the anterolateral wall, a typical non-ischemic pattern of injury. These findings are consistent with inflammatory myocardial involvement, commonly seen in viral or immune-mediated myocarditis. (**b**): Cardiac magnetic resonance (CMR) image demonstrating ischemic cardiomyopathy. The image reveals marked apical thinning with transmural late gadolinium enhancement (LGE) in the left anterior descending (LAD) artery territory, consistent with chronic infarction and scar formation. These findings are typical of prior anterior myocardial infarction with associated adverse remodeling. (**c**): 4-ch delayed enhancement CMR in apical HCM showing mid-septal LV hypertrophy and apical scar. (**d**): Short-axis LGE image revealing inferolateral mid-myocardial to epicardial lateral wall hyper-enhancement and concentric LV hypertrophy in Fabry’s disease. (**e**): Cardiac magnetic resonance imaging (CMR) in the 4-chamber view with late gadolinium enhancement (LGE) in a patient with cardiac amyloidosis. The image demonstrates diffuse, global subendocardial, and transmural late gadolinium enhancement, a hallmark feature of cardiac amyloid infiltration. Note the difficulty in nulling the myocardial signal, another characteristic finding in cardiac amyloidosis. (**f**): Cardiac magnetic resonance (CMR) image in a patient with endomyocardial fibrosis (EMF). The four-chamber late gadolinium enhancement (LGE) image demonstrates apical scarring with superimposed thrombus in the left ventricle, characteristic of EMF, and reflects chronic fibrotic remodeling leading to restrictive physiology. (**g**): T2* relaxation curve showing exponential signal decay over time, characteristic of myocardial iron overload. The yellow line represents the T2 relaxation process, which describes the decay of transverse magnetization due to interactions between nearby spins. The black line shows the T1 relaxation process, which depicts the recovery of longitudinal magnetization as spins realign with the external magnetic field. These curves are fundamental for differentiating tissue types in MRI based on their distinct relaxation times. The rapid drop in signal intensity reflects shortened T2* values due to increased magnetic susceptibility from iron deposition. Quantitative T2* analysis is essential for assessing the severity of cardiac siderosis, particularly in conditions such as thalassemia major or hemochromatosis. (**h**): LV cine image displaying prominent trabeculations and deep intertrabecular recesses, predominantly in the apical and lateral segments characteristic of left ventricular non-compaction (LVNC). (**i**): Short-axis cardiac magnetic resonance (CMR) image of the mid-left ventricle used for T2* mapping. The green line represents the outer boundary of the myocardial wall, while the red line indicates the inner endocardial lining. The region of interest (ROI) (in yellow) is typically placed in the interventricular septum to quantify myocardial T2* relaxation times. This technique is essential for detecting and monitoring myocardial iron overload, with lower T2* values indicating increased iron deposition.

**Figure 7:**
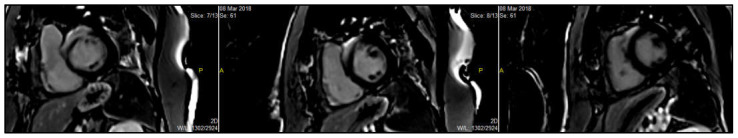
CMR image of a patient with cardiac sarcoidosis. Short-axis CMR image showing mid left ventricular patchy late gadolinium enhancement (LGE) in the basal septum and anterior wall with RV involvement, suggestive of cardiac sarcoidosis. (In the image, P = posterior; A = anterior).

Peripartum cardiomyopathy (PPCM) is a rare but potentially life-threatening form of heart failure that occurs toward the end of pregnancy or in the early postpartum period, typically within the last month of pregnancy and up to five months after delivery. It is characterized by left ventricular systolic dysfunction, often with a reduced ejection fraction (usually <45%), in women without a prior history of heart disease. The exact cause remains unclear, but proposed mechanisms include autoimmune, inflammatory, and vascular factors [61].

Cardiac magnetic resonance (CMR) plays an important role in the evaluation of PPCM, particularly when the diagnosis is uncertain or when other potential etiologies need to be excluded. Key roles of CMR include assessment of cardiac structure and function. CMR provides accurate and reproducible quantification of ventricular volumes, ejection fraction, and wall motion abnormalities, which are essential in diagnosing PPCM. CMR can detect myocardial edema, inflammation, and fibrosis using T1/T2 mapping and late gadolinium enhancement (LGE). PPCM typically shows little or no LGE, helping distinguish it from other causes of cardiomyopathy, such as myocarditis or ischemic heart disease. CMR helps rule out other conditions that may mimic PPCM, including inflammatory myocarditis, Takotsubo cardiomyopathy, or ischemic injury, especially in women with atypical presentation. Lastly, the absence of LGE on CMR is associated with a better prognosis and higher likelihood of recovery in PPCM, whereas the presence of fibrosis may indicate a more persistent form of dysfunction [61].

CMR has emerged as a powerful tool for arrhythmic risk stratification in patients with ischemic and non-ischemic (dilated) cardiomyopathies, providing detailed tissue characterization that goes beyond conventional metrics like left ventricular ejection fraction (LVEF) [55].

A key advantage of CMR is its ability to detect and quantify myocardial fibrosis using late gadolinium enhancement (LGE). Fibrosis acts as a substrate for ventricular arrhythmias by promoting electrical heterogeneity and reentry circuits. In ischemic cardiomyopathy, LGE identifies infarct-related scar and border zones, both of which correlate with ventricular tachyarrhythmia risk. In dilated cardiomyopathy (DCM), the presence and pattern of mid-wall or patchy LGE are strong, independent predictors of sudden cardiac death (SCD), even in patients with relatively preserved LVEF. Numerous studies have shown that the presence and extent of LGE are more predictive of arrhythmic events than LVEF alone [55].

Current guidelines recommend ICD implantation primarily based on reduced LVEF (≤35%), but this approach lacks sensitivity and specificity. CMR can help refine this decision. Patients with fibrosis on LGE-CMR, regardless of LVEF, may benefit from ICD due to higher SCD risk. Conversely, those without LGE, even with reduced LVEF, may have a lower arrhythmic risk, potentially avoiding unnecessary device implantation. CMR also assists in cardiac resynchronization therapy (CRT) planning by identifying scar location, LV dyssynchrony, and optimal lead positioning, all of which improve CRT response [62].

Emerging CMR techniques such as T1 mapping and extracellular volume (ECV) quantification allow for the assessment of diffuse interstitial fibrosis, offering potential for arrhythmic risk stratification even in the absence of overt LGE. Its integration into clinical workflows may lead to more personalized and precise heart failure management, particularly as evidence continues to build in support of its prognostic utility [63].

Emerging cardiac MRI techniques like GLS quantification using feature tracking analysis can detect early myocardial impairment and subclinical systolic dysfunction, making them particularly valuable in HFpEF and for risk stratification [64]. Myocardial tissue characterization can be carried out without the need for contrast in native T1 and T2 mapping. Native T1 mapping is useful for detecting diffuse fibrosis, edema, or infiltration (e.g., amyloidosis), while T2 mapping reflects myocardial edema and inflammation, which can be important in myocarditis or acute decompensated heart failure. Derived from T1 mapping before and after contrast administration, ECV estimates the proportion of myocardium composed of extracellular matrix. Elevated ECV indicates diffuse myocardial fibrosis, a key pathophysiological component in both HFrEF and HFpEF, and is associated with adverse outcomes. Together, these biomarkers are increasingly used in research and clinical practice to refine diagnosis, guide therapy, and predict prognosis in heart failure. However, standardization of techniques and broader accessibility remain important next steps [63,64].

## 7. Role of Cardiac CT in Heart Failure

**Figure 8:**
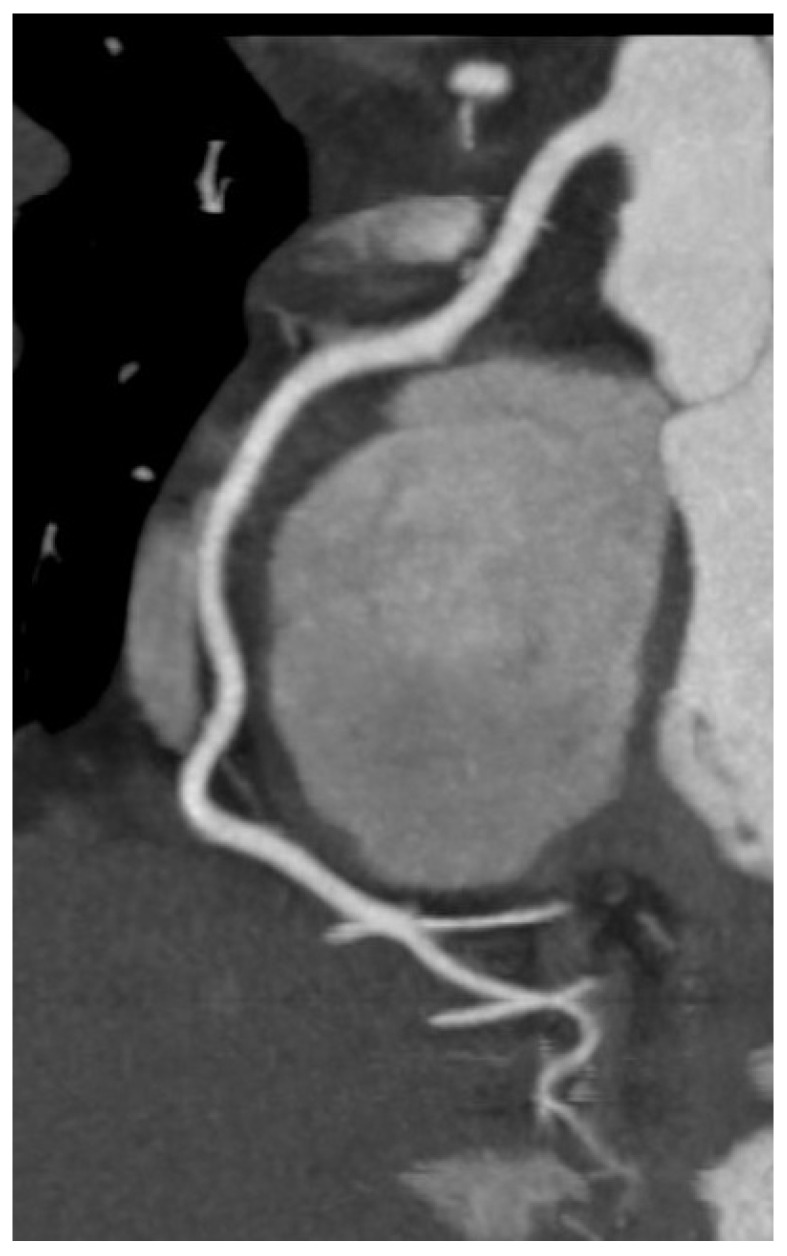
Coronary CT angiography (CCTA) image showing course of normal right coronary artery (RCA). CCTA image demonstrating normal right coronary artery (RCA) in a patient evaluated for chest pain and inferior ST-T changes on ECG.

Cardiac computed tomography (CT) assumes an increasingly significant role in the diagnostic evaluation and management of HF, particularly when there is a necessity to assess coronary anatomy, structural heart disease, or pericardial pathology. Coronary CT angiography (CCTA), a non-invasive, very sensitive technique is the major use of cardiac CT in HF for identifying or ruling out coronary artery disease (CAD), a major cause of HF. Among patients with low to intermediate pre-test probability of CAD, CCTA has a great negative predictive value and can reliably rule out obstructive lesions with great accuracy (Figure 8) [65,66].

Apart from coronary imaging, cardiac CT is increasingly used for pre-procedural planning, particularly in patients for transcatheter aortic valve implantation (TAVI), mitral valve interventions, or pulmonary vein isolation for atrial fibrillation [67]. For HF patients with valvular disease, CT enables a thorough evaluation of vascular access plan, leaflet morphology, and annular size.

Particularly when MRI is contraindicated or unavailable, CT helps for exact assessment of cardiac and extracardiac structures including ventricular volumes, wall thickness, and calcifications. In cases of suspected constrictive pericarditis, CT aids in the differentiation from restrictive cardiomyopathy by visualizing pericardial thickening and calcification [68,69].

Dual-energy CT and myocardial perfusion CT advancements help in the detection of myocardial perfusion and extracellular volume, thereby bridging the gap toward tissue characterization ordinarily restricted to cardiac MRI [70].

Even with these benefits, cardiac CT suffers limitations including radiation exposure, dependence on iodinated contrast (which is worrisome regarding renal insufficiency), and reduced soft tissue definition relative to MRI. Nevertheless, in the right clinical setting, cardiac CT presents a useful supplementary tool in the comprehensive assessment of HF that offers fast, detailed anatomical information impacting diagnostic and therapeutic avenues.

## 8. Role of Nuclear Imaging and PET in Heart Failure

**Figure 9:**
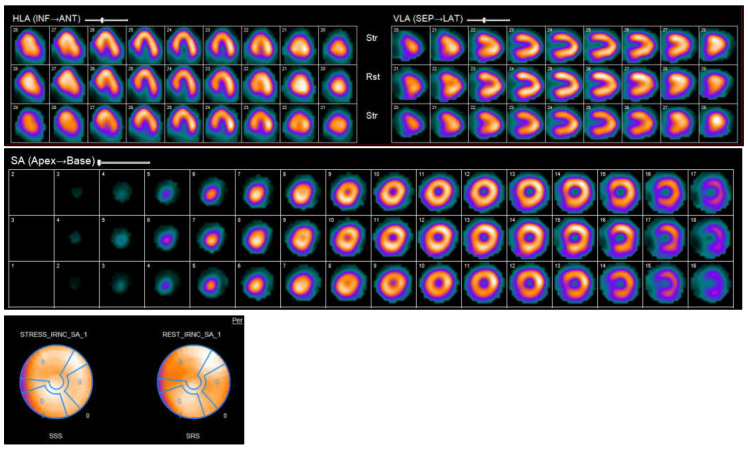
SPECT myocardial perfusion imaging shows distribution of myocardial perfusion in left ventricle. (i): SPECT myocardial perfusion imaging in the long-axis (LAX) view. The image demonstrates tracer distribution along the left ventricular myocardium, allowing assessment of regional uptake patterns. This view is essential for evaluating anterior, septal, lateral, and inferior wall involvement in various cardiac pathologies during rest and stress. (ii): SPECT myocardial perfusion imaging in the short-axis (SAX) view. The image displays cross-sectional slices of the left ventricle from base to apex, enabling detailed evaluation of regional tracer uptake. This view is critical for assessing perfusion defects, scar tissue, or abnormal myocardial uptake patterns in conditions such as ischemic heart disease or cardiac amyloidosis. (iii): Comparison of stress and rest SPECT myocardial perfusion bull’s eye plots. The plots illustrate regional tracer uptake in the left ventricle, segmented into 17 standard regions. Changes in relative counts between stress and rest studies are used to assess reversible or fixed myocardial defects.

Nuclear imaging techniques, such as SPECT and PET, provide unique insights into the pathophysiology, blood flow, viability, and metabolic condition of the myocardium in cases of HF. These imaging techniques are particularly useful for identifying the root cause of HF, supporting revascularization strategies, and assessing inflammatory or infiltrative conditions. Myocardial perfusion imaging (MPI) through SPECT or PET is commonly utilized to evaluate ischemia and viability in patients with HF that is suspected to be of ischemic origin. PET provides enhanced spatial and temporal resolution, and when used alongside fluorodeoxyglucose (FDG) imaging, it can distinguish between viable, hibernating myocardium and scar tissue—an essential factor in determining the potential advantages of revascularization in ischemic cardiomyopathy (Figure 9) [71,72].

**Figure 10:**
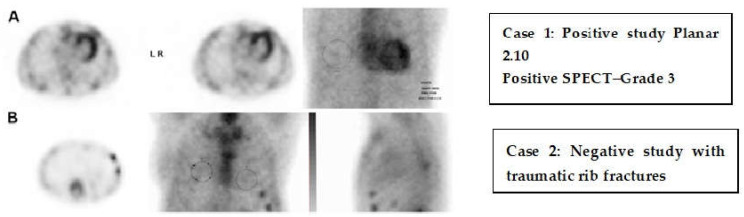
Comparative SPECT-CT scan images in evaluation of cardiac amyloidosis. (**A**): Positive SPECT-CT scan in a patient with transthyretin (ATTR) cardiac amyloidosis. The fused SPECT-CT images demonstrate intense myocardial uptake of technetium-99m-labeled pyrophosphate (^99^ᵐTc-PYP), with myocardial tracer activity equal to or greater than bone uptake (Perugini Grade 2–3). This finding is highly specific for ATTR amyloidosis in the absence of monoclonal protein. (**B**): Negative SPECT-CT study images in a patient with rib fractures. The images show focal tracer uptake corresponding to areas of traumatic rib injury, with no abnormal myocardial uptake. This pattern is consistent with trauma and demonstrates a lack of cardiac uptake, excluding transthyretin (ATTR) cardiac amyloidosis.

FDG-PET is crucial for detecting cardiac sarcoidosis because of its ability to highlight active inflammation in the myocardium, particularly when used in addition to perfusion tracers that demonstrate a discrepancy between FDG uptake and perfusion defects [73]. Serial PET scans are often beneficial for assessing response to treatment. In cases of cardiac amyloidosis, bone-targeting SPECT tracers such as 99mTc-pyrophosphate (PYP) or 99mTc-DPD are useful to differentiate transthyretin (ATTR) amyloidosis from light-chain (AL) amyloidosis, showing significant diagnostic accuracy, especially with cardiac uptake in the absence of monoclonal proteins [74] (Figure 10). This approach has become a vital non-invasive diagnostic tool, reducing the need for endomyocardial biopsy. Newly developed PET tracers also facilitate the measurement of myocardial blood flow and coronary flow reserve, which can unmask microvascular dysfunction as one of the contributing factors in patients with HFpEF [75]. Additionally, novel PET agents targeting fibrosis and sympathetic innervation are under investigation for better phenotyping and risk stratification in HF patients [76].

Positron emission tomography (PET) evaluates both perfusion and metabolic activity—typically using fluorodeoxyglucose (FDG)—allowing precise differentiation between viable and scarred myocardium. It is highly sensitive and specific but less widely available and more expensive. Single-photon emission computed tomography (SPECT) is more accessible but less accurate than PET, with lower resolution and potential underestimation of viability [71,72].

Although nuclear imaging has a lower spatial resolution than CT or MRI, it offers metabolic and molecular information that is not available via other techniques. Nonetheless, drawbacks of the technique include a limited supply of advanced PET tracers, exposure to radiation, higher costs, and inaccessibility in rural settings [71,72].

## 9. Recent Advances and Emerging Imaging Modalities in Heart Failure

Beyond structural evaluation, advancements in cardiovascular imaging have greatly improved the diagnosis, phenotyping, and prognostication of HF by offering thorough functional, metabolic, and molecular data. These innovations support the paradigm shift toward precision medicine in HF.

Three-dimensional echocardiography and strain imaging, especially global longitudinal strain (GLS), have become paramount for early detection of subclinical myocardial dysfunction, even in the presence of preserved ejection fraction [77]. Automated and AI-assisted echocardiographic analysis is expanding reproducibility and permitting point-of-care decision-making.

With sophisticated tissue mapping methods including T1, T2, and T2* mapping as well as extracellular volume (ECV) quantification, CMR continues to evolve further and enable better non-invasive identification of fibrosis, edema, infiltration, and iron overload with great diagnostic accuracy [78]. Novel contrast agents and faster imaging protocols are expanding its clinical utility.

Cardiac CT is being integrated with functional data through CT-derived fractional flow reserve (FFR-CT) and myocardial perfusion CT, thereby providing a combined anatomical and physiological assessment in patients with ischemic HF [79]. Dual-energy CT and photon-counting CT are under investigation for improved tissue contrast and lower radiation doses [80].

Emerging PET tracers targeting myocardial inflammation (e.g., 68Ga-DOTATATE), sympathetic innervation (e.g., 11C-HED), and fibrosis (e.g., 68Ga-CBP8) provide insights into the molecular basis of HF in nuclear imaging, potentially guiding personalized therapy [81]. Quantification of myocardial blood flow (MBF) and coronary flow reserve (CFR) using PET is helping to unmask microvascular dysfunction in HFpEF [82].

Artificial intelligence (AI) and machine learning are revolutionizing imaging analysis by enabling automated segmentation, risk prediction, and pheno-group classification across imaging modalities. Artificial intelligence (AI)-driven image analysis is transforming heart failure diagnosis and management by enhancing the precision, speed, and consistency of cardiac imaging interpretation. In heart failure, AI algorithms—especially those using machine learning and deep learning—can automatically quantify cardiac structures (e.g., chamber volumes, ejection fraction), detect subtle functional abnormalities (such as impaired strain patterns), and identify imaging biomarkers associated with different heart failure phenotypes, including HFpEF. AI also enables pattern recognition across multimodal imaging data (e.g., echocardiography, MRI, CT), supporting earlier detection, risk stratification, and potentially more personalized treatment approaches. However, integration into clinical workflows and validation across diverse populations remain ongoing challenges. These tools are being integrated into clinical workflows to stratify risk and tailor therapies [83].

Recent studies have demonstrated the huge potential of generative AI (Gen AI) to enhance HF imaging and diagnosis. One such study utilizing the Multi-Ethnic Study of Atherosclerosis (MESA) dataset revealed that AI-CAC, a process in which artificial intelligence is applied on coronary artery calcium (CAC) scans, is better than traditional biomarkers like NT-proBNP and Agatston CAC score for the prediction of first heart failure within 15 years. The AI-CAC model had an area under the receiver operating characteristic curve (AUC) of 0.826, much higher than NT-proBNP (0.742) and the Agatston score (0.712), indicating its superior predictive ability [84]. Researchers introduced a conditional Generative Adversarial Network (cGAN) named echoGAN, which was designed to expand the field of view (FoV) in transthoracic echocardiography (TTE) with high resolution. This innovation allows for a better comprehensive understanding of cardiac anatomy, which can make diagnosis more precise and reduce the learning curve of clinicians [85]. A study that integrated clinical data, stress test findings, and SPECT/CT myocardial perfusion imaging parameters developed an AI model predicting hospitalization for acute heart failure exacerbation. The model demonstrated an AUC of 0.87, better than traditional approaches and enabling earlier interventions to prevent HF hospitalization [86]. Researchers at UT Southwestern Medical Center developed a machine learning model that can diagnose patients with diabetic cardiomyopathy—a condition that renders a patient prone to heart failure. This device offers a data-based approach for the detection of high-risk phenotypes, which allows early intervention and precision prevention [87]. A novel paradigm combining video, text, and clinical information with large language models was introduced to assess heart failure. The multi-modal system mimics the process of doctor–patient consultation, allowing a comprehensive assessment and enhanced treatment plan, and has proved to be more accurate than single-modal AI models [88]. These studies highlight the revolutionary potential of generative AI in heart failure imaging and diagnosis, offering enhanced predictive capability, improved diagnostic accuracy, and personalized treatment plans.

Moreover, hybrid imaging (e.g., PET/MRI) is emerging as a comprehensive platform for combining anatomic, functional, and molecular data in a single session, though its clinical use is currently limited to specialized centers [89].

These innovations are transforming the landscape of HF imaging, allowing earlier diagnosis, refined phenotyping, and targeted interventions. As technology advances, future research should focus on standardization, cost-effectiveness, and integration into broader HF management strategies.

## 10. Conclusions

This review emphasizes the most recent developments in cardiac imaging and their application in the diagnosis and management of heart failure.

Echocardiography serves as the primary imaging modality, particularly due to its portability, even within the intensive care setting.

It may be supplemented by additional modalities, selected based on their capacity to address specific clinical inquiries while considering contraindications and risks associated with particular tests. The accuracy of clinical outcomes and the management strategy significantly relies on imaging modality, as well as the experience of the operator and the center. Appropriate imaging modality needs to be utilized as per the current guidelines and the clinical decision algorithms acknowledging the benefits and pitfalls of each imaging technique for the optimum and precise diagnosis and management of heart failure patients. Recent advances include AI-driven image interpretation, 3D echocardiography, photon-counting CT, molecular PET tracers, and hybrid imaging technologies (e.g., PET/MRI), all contributing to more precise and personalized HF care.

Current drug therapy for heart failure (HF) is tailored to the specific phenotype, with the most robust evidence in heart failure with reduced ejection fraction (HFrEF). Guideline-directed medical therapy (GDMT) includes four foundational classes: angiotensin receptor–neprilysin inhibitors (ARNIs)**,** beta-blockers, mineralocorticoid receptor antagonists (MRAs), and sodium-glucose co-transporter 2 (SGLT2) inhibitors—all of which improve survival and reduce hospitalizations. In heart failure with preserved ejection fraction (HFpEF), SGLT2 inhibitors are currently the only class with consistent benefits, while MRAs and ARNIs may offer modest improvements in selected patients. Diuretics are used across all HF types for symptom relief [90]. Ongoing research continues to refine and personalize pharmacological approaches based on patient-specific pathophysiology and comorbidities.

In conclusion, the integration of structural, functional, and molecular imaging is transforming the landscape of heart failure evaluation. Clinicians should tailor imaging strategies based on clinical context, availability, and patient-specific considerations, while staying informed about emerging technologies that promise to improve diagnosis, guide therapy, and enhance prognostic precision.

## Figures and Tables

**Table 1:** Global incidence and prevalence of heart failure and summary of key characteristics [3,4].

Region/Country	Prevalence	Incidence	Key Characteristics
Global	~64 million people	Varies by region	Increasing due to aging populations and improved survival post-MI
United States	~6.7 million adults	~10 per 1000 individuals/year	Leading cause of hospitalization in ≥65 years; projected to reach 8 million by 2030
Europe	1–2% general population	~3–5 per 1000 individuals/year	Prevalence ≥ 10% in people over 70; substantial healthcare burden
Sub-Saharan Africa	~9.4 million cases	High (exact data limited)	Common causes include hypertension, rheumatic heart disease, and cardiomyopathies
South Asia	~2–3% urban adults	Increasing	Mixed etiology: ischemic, hypertensive, rheumatic; earlier onset in low-income regions
Latin America	~1–2%	Moderate to high	Rapid urbanization and aging contribute to increasing prevalence

**Table 2:** Etiologies of heart failure (arranged by pathophysiological category).

Category	Etiology
Ischemic Causes	Coronary artery disease (CAD) [5]
Pressure Overload	Hypertension [6]
Volume Overload	Valvular heart disease (e.g., aortic or mitral regurgitation/stenosis) [7]
Myocardial Disease	Cardiomyopathies: dilated, hypertrophic, restrictive, e.g., EMF [8]
Inflammatory Causes	Myocarditis (often viral or autoimmune) [9]
Metabolic/Endocrine Disorders	Diabetes mellitus, thyroid disorders (hyper-/hypothyroidism), obesity [10,11]
Toxic Causes	Alcohol abuse, chemotherapy (e.g., anthracyclines), cocaine [12,13]
Genetic Causes	Familial cardiomyopathies, inherited channelopathies [14]
Infiltrative Diseases	Amyloidosis, sarcoidosis [15,16]
Peripartum	Peripartum cardiomyopathy [17]
Congenital	Congenital heart defects (e.g., septal defects) [18]
Arrhythmia-Related	Chronic tachyarrhythmias (e.g., atrial fibrillation), bradyarrhythmia [19]
Nutritional Deficiencies	Thiamine deficiency (beriberi), selenium deficiency [20]

**Table 3:** An overview of current imaging modalities in HF.

Imaging Modality	Diagnostic Accuracy *	Availability	Cost	Key Clinical Indications
Transthoracic Echocardiography (TTE)	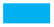 (4/5)	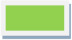	+	Initial HF assessment, LVEF measurement, valve function, pericardial disease
Speckle-Tracking Echocardiography (GLS)	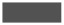 (5/5)	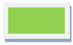	++	HFpEF, early systolic dysfunction, chemotherapy monitoring
Cardiac MRI (CMR)	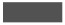 (5/5)	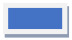	++++	Tissue characterization (fibrosis, infiltration), ventricular volumes, congenital HF, viability assessment
Cardiac CT	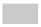 (3.5/5)	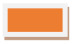	+++	Coronary artery disease assessment, structural planning (e.g., TAVI), pericardial disease
Nuclear Imaging (SPECT/PET)	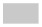 (3.5/5)	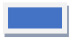	++++	Myocardial perfusion, viability, sarcoidosis, amyloidosis
Stress Echocardiography	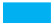 (4/5)	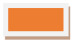	++	Ischemic evaluation, exercise-induced diastolic dysfunction (HFpEF)

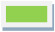
 Widely available in most centers. 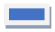 Limited availability in only specialized centers. 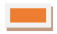 Moderate availability in most centers. +, ++: cheap; +++: relatively costly; ++++: expensive. * Note: Shading intensity reflects the magnitude of the accuracy, with darker shades representing better diagnostic accuracy.

**Table 4:** 2021 ASE/EACVI diastolic function guidelines key parameters [37].

Echocardiographic Parameter	Abnormal Threshold	Interpretation
E/e′ ratio (average septal/lateral)	>14	Elevated LV filling pressures
Septal e′ velocity	<7 cm/s	Impaired myocardial relaxation
Lateral e′ velocity	<10 cm/s	Impaired myocardial relaxation
LA volume index (LAVI)	>34 mL/m^2^	Chronic elevation in LV filling pressures
Peak TR velocity	>2.8 m/s	Suggestive of pulmonary hypertension

A diagnosis of diastolic dysfunction is supported if ≥3 of the 4 parameters are abnormal.

**Table 5:** Strain imaging in HFpEF: [38].

Parameter	Findings in HFpEF
GLS	Often normal to mildly reduced (−16% to −18%)
Left atrial strain (reservoir strain)	<23% indicates increased LA stiffness and elevated LV filling pressure [38]
LV early diastolic strain rate	Reduced in HFpEF (optional parameter)

**Table 6:** Diagnostic criteria for cardiotoxicity using strain imaging. Based on guidelines from ASE, EACVI, and ESC cardio-oncology consensus documents [44,45,46]:

Parameter	Criteria for Cardiotoxicity
Baseline GLS	Obtain before initiating therapy for reference
Relative reduction in GLS	≥15% relative reduction from baseline suggests early subclinical cardiotoxicity
Absolute GLS threshold	No universal cutoff, but GLS less negative than −18% often considered abnormal
LVEF change	≥10% decrease to a value < 53% = cardiotoxicity (per ASE/EACVI)
Combined assessment	Relative GLS drop ≥ 15% with normal LVEF = subclinical LV dysfunction
Recovery monitoring	Persistent GLS abnormalities may indicate incomplete myocardial recovery or ongoing damage

**Table 7:** Echo and strain imaging features in nonischemic cardiomyopathy.

Cardiomyopathy	Echocardiographic Features	Strain Imaging Features
Dilated (DCM)	- LV dilation - ↓ LVEF - Global hypokinesia	- Markedly ↓ GLS - ↓ RV strain - ↓ LA strain if chronically elevated pressures
Hypertrophic (HCM)	- Asymmetric septal hypertrophy - SAM of mitral valve - Normal or ↑ LVEF	- ↓ GLS in hypertrophied segments - Preserved RV strain early - ↓ LA strain (diastolic dysfunction)
Restrictive (RCM)	- Normal/mildly reduced LV size - Biatrial enlargement - Grade III diastolic dysfunction	- Mild ↓ GLS - Markedly ↓ LA strain - ↓ RV strain if RV involved
Amyloidosis	- Increased wall thickness - Granular “sparkling” appearance - Small pericardial effusion	- Severely ↓ GLS with apical sparing (“cherry-on-top”) - Severely ↓ LA strain - ↓ RV strain
Sarcoidosis	- Segmental wall motion abnormalities specifically in basal septum - RV involvement possible	- Focal ↓ GLS (often basal) - ↓ RV strain if RV involved
Arrhythmogenic RV (ARVC)	- RV dilation - Regional RV wall motion abnormalities	- ↓ RV free wall strain - GLS typically preserved early
LV Noncompaction (LVNC)	- Prominent trabeculations - Noncompacted: compacted ratio > 2.3	- Segmental ↓ GLS - ↓ strain in noncompacted segments
Myocarditis	- Normal or regional wall motion abnormalities in non-coronary distribution - Possible pericardial effusion	- Regional ↓ GLS - ↓ strain can precede EF drop

**Table 8:** Key echocardiographic parameters in RHF [48,49].

Parameter	Abnormal Threshold/Significance
RV basal diameter	>42 mm (dilated RV)
RV mid-cavity diameter	>35 mm
RV/LV ratio (end-diastole)	>1 (RV enlargement)
Tricuspid Annular Plane Systolic Excursion (TAPSE)	<17 mm = RV systolic dysfunction
RV Fractional Area Change (FAC)	<35% = reduced RV systolic function
S′ (Tissue Doppler at tricuspid annulus)	<9.5 cm/s = RV systolic dysfunction
RA area (end-systole)	>18 cm^2^ = RA enlargement
IVC diameter and collapse	>21 mm with <50% collapse = elevated RA pressure

**Table 9:** Key echocardiographic clues for PAH (per ESC Guidelines) [50].

Parameter	Threshold/Interpretation
Peak TR velocity	>2.8 m/s = elevated pulmonary artery pressure
Estimated PASP	>35–40 mmHg (may exceed 60–70 mmHg in advanced PAH)
PA/Ao ratio (main pulmonary artery to aortic root)	>1 = suggestive of PAH
RV outflow acceleration time	<105 ms = increased pulmonary pressure
Interventricular septal flattening	“D-shaped” LV = RV pressure overload
Pulmonary artery dilation	>25–29 mm (main PA)
Right atrial enlargement	Supports chronic pressure overload

**Table 10:** Speckle-tracking strain imaging in RHF/PAH [51,52].

Strain Parameter	Significance/Normal Threshold
RV free wall longitudinal strain (RV FWLS)	>−20% = normal; <−20% = impaired RV function
RV global longitudinal strain (RV GLS)	May underestimate dysfunction compared to RV FWLS
Right atrial strain	Decreased reservoir strain = elevated RA pressure, poor prognosis
Septal strain patterns	Flattening/inversion indicates interventricular dependence

## Data Availability

No new data was created.

## Abbreviations

The following abbreviations are used in this manuscript:

ACEI	Angiotensin converting enzyme inhibitors
ARNI	Angiotensin receptor/neprilysin inhibitor
ARVC	Arrhythmogenic right ventricular cardiomyopathy
BNP	B type natriuretic peptide
CAD	Coronary artery disease
CCTA	Coronary cardiac tomography angiogram
CMR	Cardiac magnetic resonance
ECV	Extracellular volume
HF	Heart Failure
HFmrEF	Heart failure with mildly reduced ejection fraction
HFpEF	Heart failure with preserved ejection fraction
HFrEF	Heart failure with reduced ejection fraction
GLS	Global longitudinal strain
LGE	Late gadolinium enhancement
LV	Left ventricle
MRA	Mineralocorticoid receptor antagonists
MPI	Myocardial perfusion imaging
MRI	Magnetic resonance imaging
PET	Positron emission tomography
STE	Speckle track echocardiography
SPECT	Single-photon emission computerized tomography
SGLT2is	Sodium glucose cotransporter-2 inhibitors
TTE	Transthoracic echocardiography
